# Engineering Solution-Processed Non-Crystalline Solid Electrolytes for Li Metal Batteries

**DOI:** 10.1021/acs.chemmater.2c03071

**Published:** 2023-01-18

**Authors:** Pooja Vadhva, Thomas E. Gill, Joshua H. Cruddos, Samia Said, Marco Siniscalchi, Sudarshan Narayanan, Mauro Pasta, Thomas S. Miller, Alexander J. E. Rettie

**Affiliations:** † Electrochemical Innovation Lab, Department of Chemical Engineering, University College London, LondonWC1E 6DH,United Kingdom; ‡ The Faraday Institution Quad One, Harwell Science and Innovation Campus, DidcotOX11 0RA,United Kingdom; § Department of Materials, University of Oxford, OX1 3PHOxford, United Kingdom

## Abstract

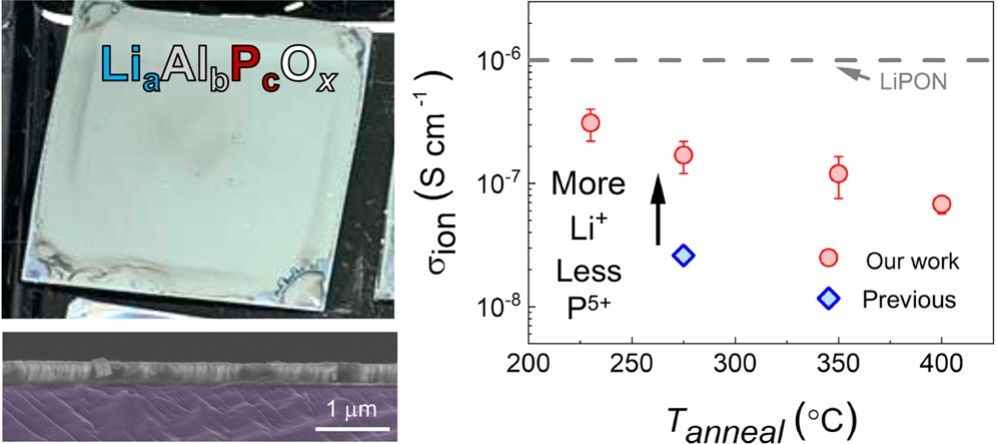

Non-crystalline Li-ion solid electrolytes (SEs), such as lithium phosphorus oxynitride, can uniquely enable high-rate solid-state battery operation over thousands of cycles in thin film form. However, they are typically produced by expensive and low throughput vacuum deposition, limiting their wide application and study. Here, we report non-crystalline SEs of composition Li–Al–P–O (LAPO) with ionic conductivities > 10^–7^ S cm^–1^ at room temperature made by spin coating from aqueous solutions and subsequent annealing in air. Homogenous, dense, flat layers can be synthesized with submicrometer thickness at temperatures as low as 230 °C. Control of the composition is shown to significantly affect the ionic conductivity, with increased Li and decreased P content being optimal, while higher annealing temperatures result in decreased ionic conductivity. Activation energy analysis reveals a Li-ion hopping barrier of ≈0.4 eV. Additionally, these SEs exhibit low room temperature electronic conductivity (< 10^–11^ S cm^–1^) and a moderate Young’s modulus of ≈54 GPa, which may be beneficial in preventing Li dendrite formation. In contact with Li metal, LAPO is found to form a stable but high impedance passivation layer comprised of Al metal, Li–P, and Li–O species. These findings should be of value when engineering non-crystalline SEs for Li-metal batteries with high energy and power densities.

## 1 Introduction

Over the past few decades, lithium-ion battery (LIB) technology has enabled a wide range of applications, principally: portable electronic devices, wearable devices and electric vehicles.^1,2^ However, with LIBs reaching maturity, a new generation of advanced batteries with increased energy and power densities are needed to usher in sweeping decarbonization and meet ambitious climate targets. Lithium metal anodes offer an ≈10× theoretical energy density compared to graphite anodes used in LIBs,^3,4^ but the use of traditional liquid electrolytes results in short cycle life as well as safety concerns due to dendrite formation in the presence of flammable solvents. Solid electrolytes (SEs) have been proposed to block dendrite propagation due to their stiffness and high transference number.^5,6^ Recently, crystalline SEs with ionic conductivity (σ_*ion*_) values rivalling liquid electrolytes have been discovered,^7^ but despite their solid form, all suffer from Li dendrite propagation at practical (dis)charge rates. In addition, unstable interphase formation and difficulty of manufacturing at scale are outstanding challenges.^4−6,8−11^

Non-crystalline SEs may uniquely meet these stringent requirements.^11−16^ One such material, lithium phosphorus oxynitride (LiPON), has demonstrated a long cycle life and high-rate operation^17−20^ in a thin-film solid-state battery (SSB). These properties have motivated work to understand its low impedance interphase and resistance to dendrite formation. Cryo-transmission electron microscopy showed that the interphase consisted of nanoscale domains of Li_2_O, Li_3_P, and Li_3_PO_4_ in an ≈100 nm thick amorphous LiPON matrix,^21^ although electrochemical techniques separately suggested an interphase thickness of 5 nm.^22^ This stable interphase has enabled its use as a Li-electrode protective coating in Li-ion, Li–sulfur, and Li–air cells.^23−25^ Regarding dendrite formation, the amorphous nature of LiPON minimizes surface roughness and grain boundaries in the bulk, which may act as sites for dendrite nucleation and growth.^26^ Recent work has also suggested that LiPON’s low electronic conductivity (σ_*e*_) may be an important aspect in its ability to block dendrites.^14^ However, applicability of LiPON at scale is limited as it is produced by costly and low throughput vacuum deposition techniques.

New non-crystalline SEs produced by scalable methods would be of great interest for advanced batteries, for example, as SEs in thin film solid state batteries or to template metal deposition in “anode-free” cells where the SE films are deposited onto the negative current collector.^12^ Solution-based processing involving direct deposition of precursor solutions followed by a moderate temperature annealing step can function as a low energy alternative to the conventional high temperature routes used for ceramic SEs.^27,28^ There have been several reports of non-crystalline SE fabrication using organic solvents in moisture-free conditions,^29−31^ but production in air using water as a solvent would be most desirable for low cost and sustainable manufacturing. Extremely smooth, dense amorphous films from aqueous solutions have long been of interest for microelectronic applications,^32^ and this framework was recently extended to Li-containing materials by Clayton et al.^33^ Although dense, smooth films were achieved, the σ_*ion*_ of these films was too low for solid electrolyte applications (≈10^–8^ S cm^–1^ vs ≈10^–6^ S cm^–1^ for LiPON).^34^

Here, we report non-crystalline Li–Al–P–O (LAPO) phases with desirable SE properties synthesized from solution. First, we systematically explore this phase space and find thin film materials with σ_*ion*_ > 10^–7^ S cm^–1^. Then, the effects of annealing temperature on the σ_*ion*_, film structure, surface roughness, and chemical composition are studied. The optimized SE is shown to exhibit a small barrier to Li-ion transport, low σ_*e*_, and mechanical properties comparable to LiPON. Finally, the electrochemical stability against Li-metal is probed and the chemical composition of the resultant interphase determined.

## 2 Experimental Methods

### 2.1 Film Fabrication

Li–Al–P–O thin films were synthesized by spin coating from aqueous precursor solutions, followed by an annealing step in air as described previously,^33^ with differences in the precursor solution compositions and the annealing temperatures used. In a typical synthesis, 50 mmol of Al(NO_3_)_3_·9H_2_O (Sigma-Aldrich) was added to 50 mL of deionized (DI) water and stirred for 1 h until completely dissolved. To this solution, 63 mmol of H_3_PO_4_ (85 wt % in water) was added and stirred overnight at 80 °C. After cooling to room temperature, 137.5 mmol of LiNO_3_ (Fisher Scientific) was added. Finally, the solution was diluted with DI water to achieve a final concentration of 0.4 M with respect to Al. This final concentration was used for all precursor solutions with the moles of LiNO_3_ or H_3_PO_4_ being varied to achieve a range of Li:Al:P ratios during compositional exploration.

Silicon (Si) substrates (p-type, boron-doped, single-side polished, resistivity < 0.1 cm, PI-KEM) were used as electrically conductive back contacts with low roughness. These were cut into 2 × 2 cm^2^ squares using a diamond scribe and sonicated separately in acetone and then IPA for 5 min, rinsing with DI water in between. Subsequently, the substrates were dried using a N_2_ gun before being O_2_ plasma-treated (Henniker HPT-100) at 100 W for 5 min to produce a hydrophilic surface.

The precursor solution was sonicated at 40 °C for 1 h and cooled to room temperature before being twice filtered using a 0.2 μm Teflon filter attached to a syringe. The solution was flooded onto the substrate, spin coated at 3000 rpm for 30 s (after a ramp rate of 6000 rpm s^–1^), and immediately transferred to a preheated hot plate at 275 °C for 1 min. The process was repeated for multilayer films, by allowing the film to cool to room temperature before spin coating the next layer. After the designated number of layers were deposited, a final anneal at the desired temperature was carried out for 1 h. For the films annealed above 275 °C, a box furnace was used with a 5 °C min^–1^ ramp rate. For the films annealed at 230 °C, the preheated hot plate was set at 230 °C so that the films were not exposed to a temperature above this value.

### 2.2 Physical Characterization

Film thickness was determined using scanning electron microscopy (LEO Gemini 1525 field emission scanning electron microscope (SEM)). For cross-sectional imaging, the brittle-fracture method was used, and a thin Au layer was sputtered to minimize charging. Multilayer films were used for ease of imaging. The film morphology and mechanical properties were characterized using atomic force microscopy (AFM, Bruker Dimension Icon with ScanAsyst) across a 10 × 10 μm^2^ film area with the average roughness calculated from 3 different areas across 1 × 1 μm^2^ using the PeakForce Quantitative Nanoscale Mechanical mode. The PeakForce tapping mode was adopted in all cases with an RTESPA-525 Si probe with reflective Al coating (Bruker Corp., *k* = 200 N m^–1^, *f*_0_ = 525 kHz). For mechanical property measurements, the probe was calibrated by the relative method, using highly oriented pyrolytic graphite (HOPG) with a nominal elastic modulus of 18 GPa for reference. At each point in the scan, alongside the morphology, the probe performed nanoindentation measurements and recorded the load and displacement of the specialized tips and cantilevers to produce a load–displacement curve. This curve was used to calculate the elastic modulus of the materials, by fitting to the Derjagin, Muller, Toropov (DMT) model.^35^ All of the results obtained by the AFM were analyzed by Nanoscope Analysis software.

### 2.3 Chemical Characterization

The film composition was determined using X-ray photoelectron spectroscopy (ThermoFisher, K-alpha XPS system, Al source) with binding energies referenced against the adventitious carbon 1s peak at 284.8 eV. The atomic percentages of each element were estimated using the peak areas and appropriate relative sensitivity factors (RSFs) from CasaXPS software for Li 1s (0.057), Al 2p (0.537), P 2p (1.192), and O 1s (2.93). A survey scan and regions around elements of interest were conducted. For lighter elements such as Li, a minimum of 30 scans were acquired.

In situ XPS coupled with Li sputtering was conducted in an in-house setup, using a Phi XPS VersaProbe III with an Al Kα X-ray source generating focused, monochromatic Al Kα X-rays at 1486.6 eV under ultrahigh vacuum conditions (the main chamber maintained at pressures between 10^–7^ and 10^–6^ Pa). Here, Li metal (3 × 3 mm^2^, 750 μm thick, Sigma-Aldrich) was attached to a sample holder within the XPS chamber, similar to the setup described by Wenzel et al., previously.^36^ The LAPO sample and Li metal were transferred to the XPS chamber using a vacuum transfer vessel directly from a glovebox to minimize air exposure. Li sputtering was conducted using an Ar^+^ ion gun, at an acceleration voltage of 4 kV and beam current of 2.8 μA, with data collected at intervals of 5 min. CasaXPS software was used to analyze the XPS data and quantify the chemical composition using Shirley background fitting. The spectra obtained prior to lithium deposition were charge corrected to adventitious C at 284.8 eV through acquired C 1s spectra. After lithium deposition the Li_2_O peak at 528.5 eV in the O 1s spectra was used for charge-correction, in accordance with the study of Wood et al.^37^

Grazing incidence X-ray diffraction (GI-XRD) was performed on a Bruker D8 Discover diffractometer with a microfocus Cu source and Vantec 500 2D detector. These films were spin coated onto fused silica substrates to minimize scattering from the substrate. The fused silica substrates were cleaned using the same procedure as the Si wafers and were purchased precut to 2 × 2 cm^2^, with a thickness of 1 mm from Multilab. The scans were performed in a theta–theta geometry with 4 frames at 120 s per frame, and the sample was rotated in the beam during collection.

### 2.4 Electronic and Electrochemical Characterization

Through-plane measurements were performed throughout. Circular Au top contact pads (1.2 mm diameter, ≈80 nm thickness) were deposited by sputtering through a shadow mask. For the bottom contact, Al foil was attached to the back of the Si substrate using conductive epoxy (Agar Scientific). An in-house cell holder was designed to take conductivity measurements, where an Au-plated screw with a rounded tip gently contacted the Au pads. In all cases 4-layer films were used as thinner films could be damaged by the screw contact pressure. The Au screw and Al back contact were connected to a potentiostat (Reference 600+, Gamry) for electrochemical measurements, with no external pressure applied other than that of the Au screw lightly touching the Au pads to make electrical contact. Note that the sputtered top contact pads defined the area of the cell (1.13 mm^2^) and were significantly larger than the rounded screw tip (≈0.20 mm^2^), so the majority of the cell was under no applied pressure. As will be shown, the relative error was relatively low over numerous σ_*ion*_ measurements on films of different compositions. Therefore, the role of contacting the contact pad and any associated pressure was not significant in this case.

Electrochemical impedance spectroscopy (EIS) was conducted using a 5 mV perturbation voltage over a frequency range of 50 Hz to 1 MHz at room temperature. The EIS data were fit using an equivalent circuit model (ECM) consisting of elementary components in a Randles type circuit.^38,39^ A resistor (*R*) and constant phase element (*CPE*) in parallel were used to model different relaxation processes, where *R*_0_ accounts for the impedance due to the ohmic resistance from electrical contacts, *R*_1_ is assigned to the bulk SE impedance (*R*_*b*_) of LAPO, and *CPE*_*w*_ accounts for the electrode polarization due to the non-symmetric blocking electrodes.^40^

The σ*_ion_* was calculated using eq 1:
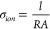
1where, *l* is the thickness of the film, *R* is the bulk SE resistance, and *A* is the geometric area. These values were averaged from 3 different films, with each film being sampled in multiple positions (>3) across the sample.

Temperature-dependent EIS measurements were conducted inside a thermal chamber in air on 3 separate samples to obtain an average. Data collection was performed during heating, and a wait time of 2 h was used at each temperature point to reach thermal equilibrium. The temperature dependence of the σ_*ion*_ was fit to an Arrhenius relationship:^41,42^
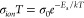
2where, *T* is temperature, σ_0_ is a pre-exponential factor dependent on temperature, *E*_*a*_ is the activation energy, and *k* is the Boltzmann constant.

For the DC polarization experiments, a voltage bias of 1 V was applied for 1 h and the current–voltage curve fit to an exponential decay function. A longer duration constant–voltage experiment was run over 12 h, which confirmed that 1 h was sufficient to reach steady state.

Finally, the electrochemical stability of LAPO with Li metal was probed by thermally evaporating Li (MBraun, EVAP) to form circular contacts (1 mm diameter, 1 μm thickness) onto a LAPO film on a Si substrate which had been dried under vacuum in a Buchi oven at 60 °C overnight, resulting in a Li|LAPO|Si configuration. We confirmed this drying process did not significantly affect the films: a room temperature σ_*ion*_ value of 1.5(7) × 10^–7^ S cm^–1^ was measured for dried Li_2.8_AlP_1.25_O_*x*_ films vs 1.8(5) × 10^–7^ S cm^–1^ with no drying step. Cu was used as the current collector, and the cell was sealed under Ar in a pouch cell bag. The cell was clamped between two plates to ensure good electrical connection. EIS was conducted at room temperature over 13 h with a Biologic MTZ-35 potentiostat, between 1 Hz and 3.7 MHz. To separate polarization contributions from the various cell components and identify all time processes in the system, a Fourier transform of the EIS data was performed for distribution of relaxation times (DRTs) analysis by^43,44^
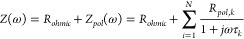
3where, *R*_*ohmic*_ is the Ohmic resistance of the SSB and is independent of frequency, while *Z*_*pol*_(ω) accounts for the polarization resistance, *R*_*pol*,*k*_ and is a function of frequency. This deconvolution is possible since the different cell processes have characteristic frequencies, and therefore time constants, associated with specific processes. A MATLAB code by Wan et al.^44^ was used to perform DRT analysis. The Li deposition and cell assembly were carried out in an Ar-filled glovebox (MBraun, <1 ppm of H_2_O and O_2_).

## 3 Results and Discussion

### 3.1 Compositional Engineering

**Figure 1:**
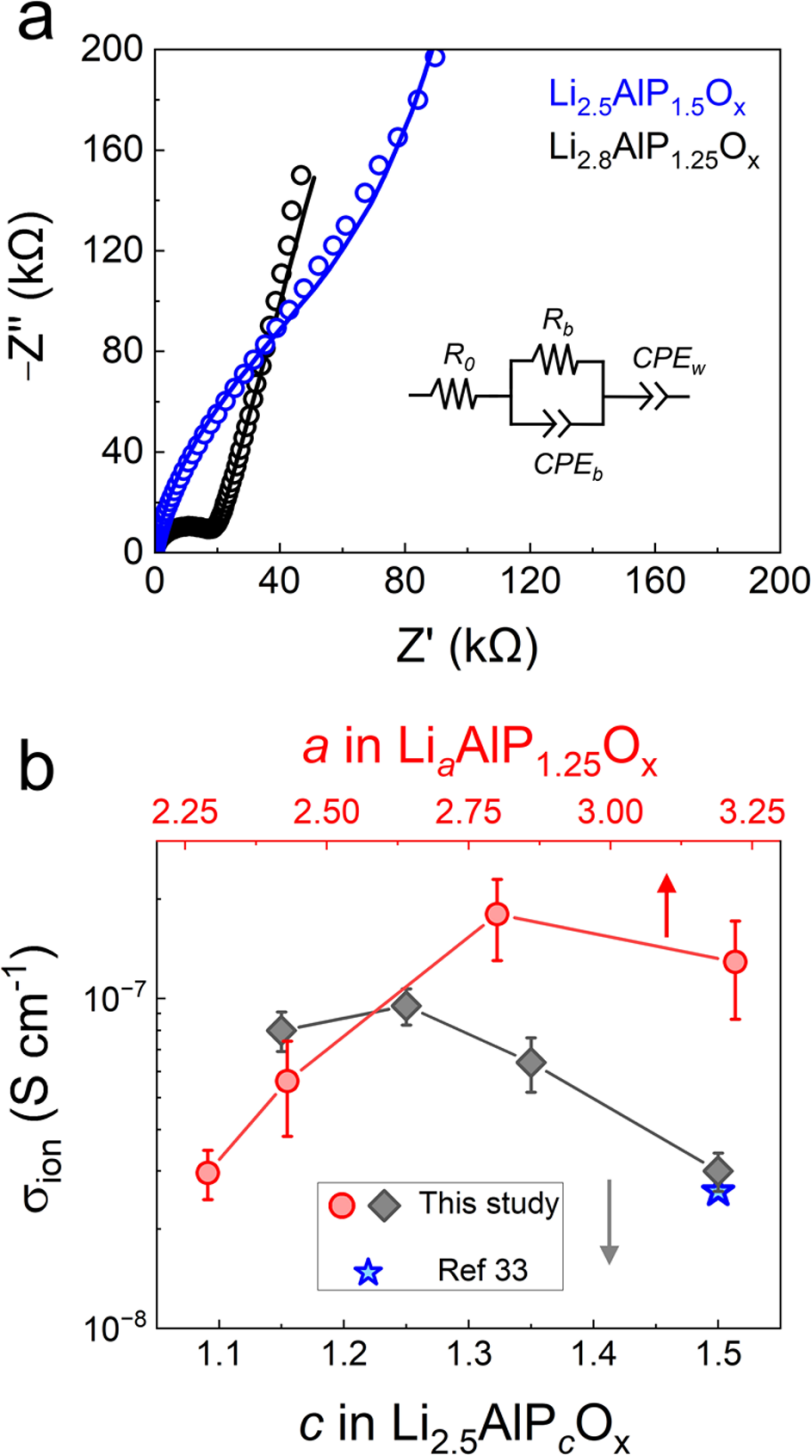
(a) EIS Nyquist plots for two LAPO compositions with the equivalent circuit model used to fit the data (inset). (b) σ_*ion*_ for various LAPO compositions determined from the fitted EIS data, points with error bars were constructed using the average ± 1 standard deviation.

Previous work^33^ determined a room temperature σ_*ion*_ value of 2.6 × 10^–8^ S cm^–1^ for the single composition Li_2.5_Al_1_P_1.5_O_5.5_ (based on the bulk glass 0.5Li_2_O–0.2Al_2_O_3_–0.3P_2_O_5_)^45^ annealed at 275 °C. Because elemental composition can strongly affect the conduction properties of SEs,^45,46^ we systematically adjusted the Li and P ratios relative to Al in Li_*a*_Al_1_P_*c*_O_*x*_ while keeping the annealing temperature constant at 275 °C. Note that *a* values were determined by XPS, while *c* represents the nominal amount of phosphorus in the precursor solutions. First, the P content in Li_2.5_Al_1_P_*c*_O_*x*_ was varied in the range 1.1 < *c* < 1.5 (Figure 1). EIS of all samples could be adequately fit with the ECM in Figure 1a (inset) which is typical for non-crystalline materials.^40^ As the P content was decreased from 1.5 to 1.25, an increase in σ_*ion*_ from 3.0(4) × 10^–8^ S cm^–1^ to 0.95(12) × 10^–7^ S cm^–1^ was observed. Values of *c* below 1.15 resulted in films with poor coverage. Fixing the optimal value of *c* = 1.25, the Li content (*a* value) was subsequently varied. By increasing *a* from 2.25 to 2.8, the σ_*ion*_ increased by almost an order of magnitude (from 3.0(5) × 10^–8^ S cm^–1^ to 1.8(5) × 10^–7^ S cm^–1^), highlighting the sensitivity of σ_*ion*_ to both Li and P content. Finally, the effect of P content in the Li-rich Li_2.8_Al_1_P_*c*_O_*x*_ was investigated, which confirmed the same optimal composition (see Figure S1 in the Supporting Information (SI)). Therefore, our initial exploration of the Li–Al–P–O phase space yielded a maximum σ_*ion*_ of 1.8(5) × 10^–7^ S cm^–1^ for the composition Li_2.8_Al_1_P_1.25_O_*x*_. To the best of our knowledge, this is the highest reported for a lithium aluminophosphate glass at room temperature (Table S1 the SI).^33,45−47^

### 3.4 Effect of Annealing Temperature

**Figure 2:**
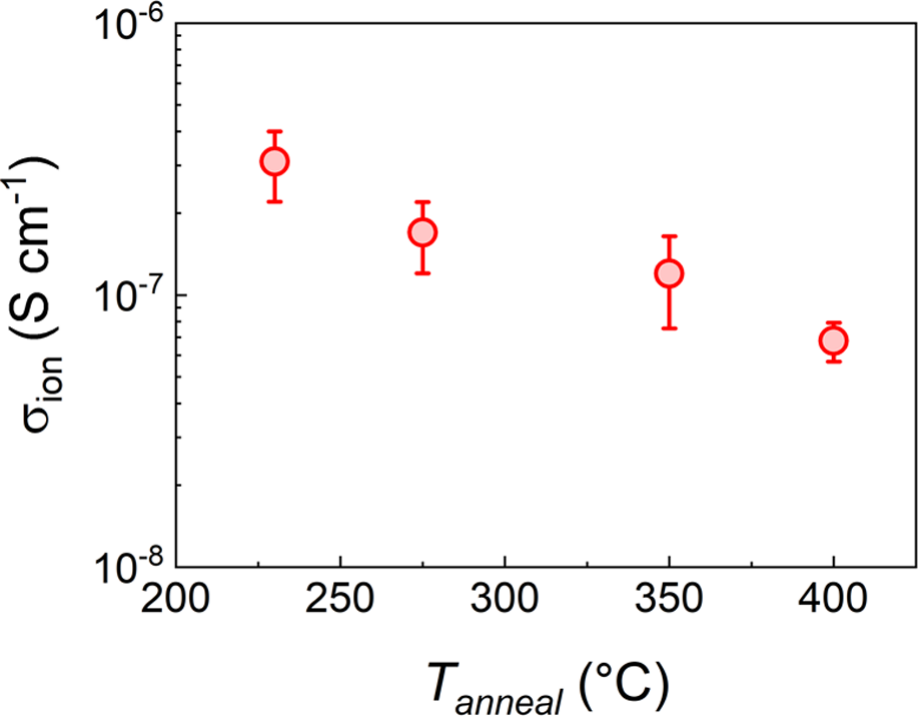
Room temperature σ_*ion*_ variation for Li_2.8_AlP_1.25_O_*x*_ films as a function of annealing temperature.

From Figure 1, the LAPO film composition with the highest σ_*ion*_ (Li_2.8_Al_1_P_1.25_O_*x*_) was chosen and the conductivity as a function of annealing temperature in air, *T*_*anneal*_, studied. An inverse relationship between σ_*ion*_ and *T*_*anneal*_ was evident, with σ_*ion*_ decreasing by a factor of ≈4 from 230 to 400 °C (Figure 2). This observation is in contrast to the work of Clayton et al.,^33^ who found negligible σ_*ion*_ (≈10^–10^ S cm^–1^) after annealing Li_2.5_Al_1_P_1.5_O_*x*_ films at 400 °C, suggesting a complex relationship between composition, annealing temperature, and film structure in this system.

The most conductive films had σ_*ion*_ values within an order of magnitude of state-of-the-art LiPON SEs (σ_*ion*_ ≈2 × 10^–6^ S cm^–1^).^34^ We note that the LiPON SEs used in SSBs are typically several micrometers in thickness, so comparable bulk resistance values would be expected for the thinner LAPO films (i.e., 100s of nm) used here. Annealing at temperatures less than 230 °C resulted in a visually spotty and inhomogeneous film appearance (Figure S2 in the SI). Crystallization may occur at higher annealing temperatures and affect ionic properties. However, laboratory GI-XRD showed no signal above the background of the fused silica substrates, indicating the films were non-crystalline at all annealing conditions investigated here (Figure S3 in the SI).

XPS was used to quantify the elemental surface composition and oxidation states of the films. Representative region scans can be located in Figure S4 in the SI. All films contained chemical species in the expected charge states: Li^+^, Al^3+^, P^5+^, and O^2–^ with a higher binding energy shoulder peak in the spectra of the latter indicative of defective oxygen or surface hydroxides.^48,49^ No N signal was detected, consistent with the loss of nitrates during annealing. Table 1 contains the surface compositions of LAPO films annealed at different temperatures. At all *T*_*anneal*_ values the measured values were in good agreement with the expected stoichiometry based on the precursor solution composition. Future work will investigate controlling the chemical inhomogeneity of the films during synthesis, e.g., by intentionally depositing layers of dissimilar composition, in addition to further exploration of the Li–Al–P–O phase space.

**Table 1:** Film Surface Composition Determined by XPS

	Stoichiometry in Li_*a*_Al_*b*_P_*c*_O_*x*_			
*T*_*anneal*_ (°C)	*a*	*b*	*c*	*x*
230	3.0	1	1.4	5.2
275	2.8	1	1.3	5.1
350	2.7	1	1.3	5.2
400	2.9	1	1.2	5.6

**Figure 3:**
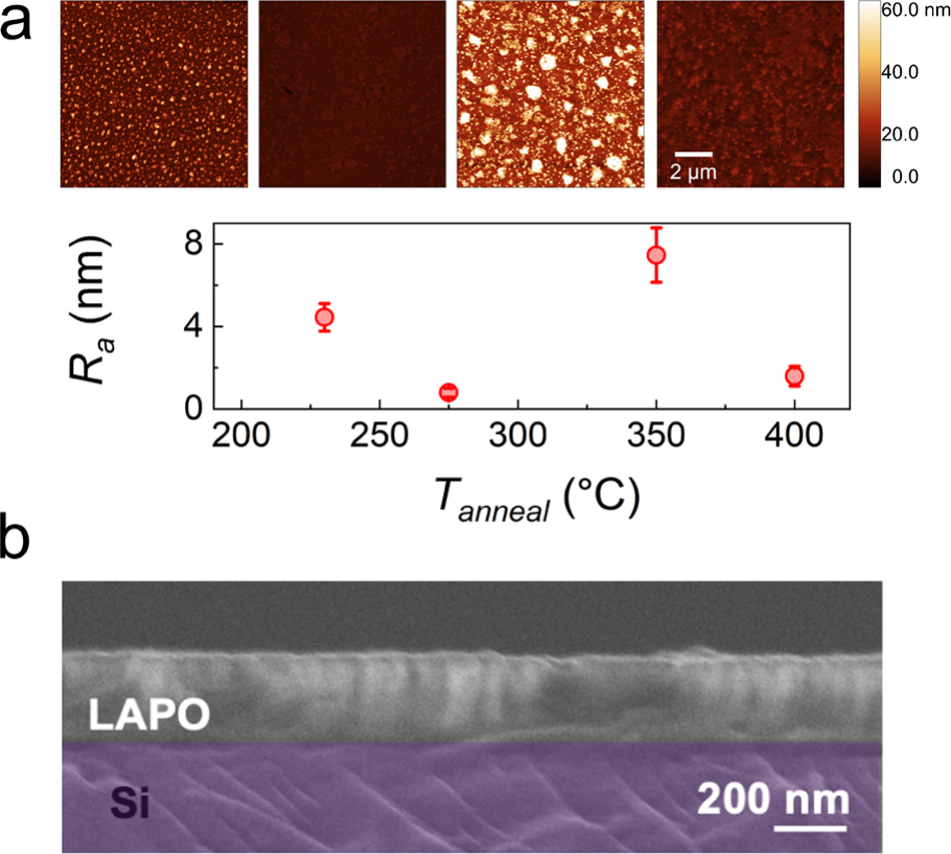
(a) AFM of the surface of single layer films and their average roughness (*R*_*a*_) as a function of annealing temperature displayed underneath. (b) Cross-sectional SEM image of a 4-layer Li_2.8_AlP_1.25_O_*x*_ film on a Si substrate annealed at 275 °C.

The film surface morphology as a function of annealing temperature was determined with AFM (Figure 3a). All films exhibited low average surface roughness (*R*_*a*_) < 10 nm, with *T*_*anneal*_ = 275 °C exhibiting the lowest *R*_*a*_ of ≈1 nm. A complex relationship was evident and reproducible across multiple samples—likely due to competing processes, such as evaporation and surface reorganization, occurring during annealing.

The Young’s modulus was determined by nanoindentation to be 54(4) GPa. This value is greater than that of sulfide SEs (≈15–20 GPa)^16,50,51^ and close to that of LiPON (77 GPa),^20^ suggestive of LAPO being sufficiently tough to suppress Li dendrite propagation. Due to the combination of low surface roughness, chemical homogeneity, and near-peak ionic conductivity, 275 °C was chosen as the optimal annealing temperature for subsequent investigations—unless otherwise indicated, the abbreviation LAPO will refer to this film composition annealed under these conditions for the remainder of the manuscript. SEM imaging confirmed these SE films were continuous and dense (Figure 3b), with a single layer thickness of ≈75 nm.

### 3.5 Activation Energy Analysis

**Figure 4:**
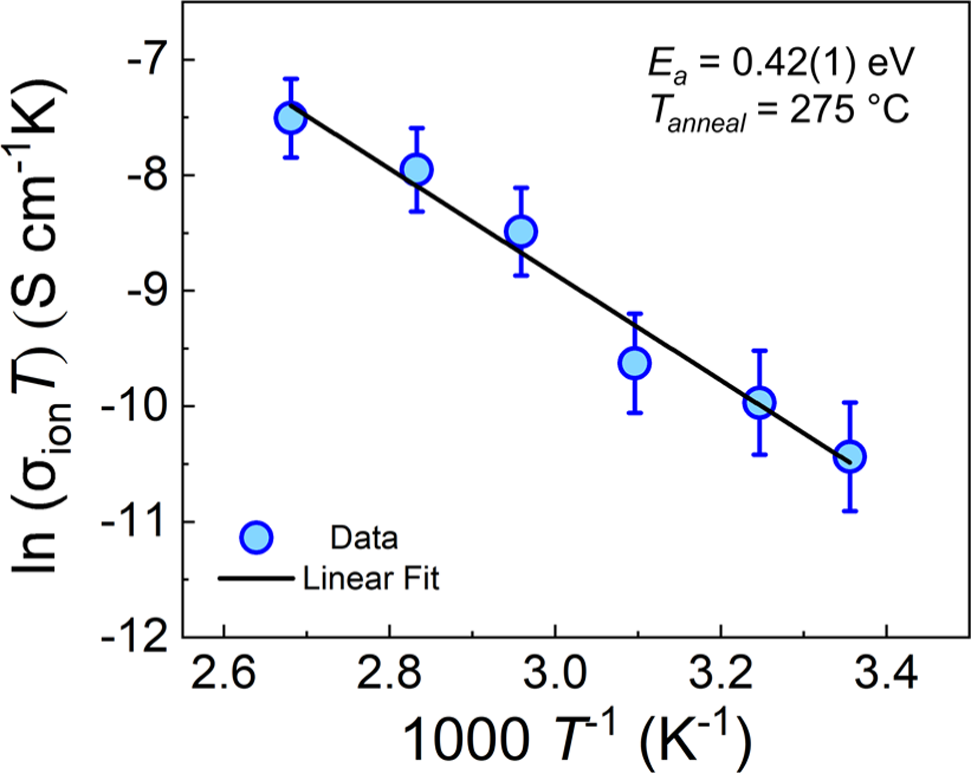
Temperature-dependent σ_*ion*_ measurements and activation energy analysis.

The activation energy, *E*_*a*_, of optimized LAPO was determined using an Arrhenius relationship (eq 2, Figure 4) as 0.42(1) eV. LAPO films annealed between 230 and 400 °C displayed activation energies in the range 0.39–0.47 eV. In general, the activation energy increased as the room temperature σ_*ion*_ decreased, consistent with more facile ion hopping leading to higher ionic conductivity (Figure S5 and Table S2 in the SI). These values are lower than those reported for thin film and bulk Li_2.5_AlP_1.5_O_5.5_ glasses, 0.67 and ≈0.6 eV, respectively,^33,45^ and even lower than that reported for LiPON (≈0.55 eV),^15^ despite the latter’s higher room temperature conductivity. This can be rationalized by considering the greater Li content of LiPON^52^ compared to LAPO, which is incorporated in the conductivity prefactor term in eq 2. Additional differences in prefactor parameters, e.g., hopping frequency, may also contribute. Returning to the other LAPO phases, additional Li in the structure could provide additional charge carriers. However, our optimized composition contains only slightly more Li (≈10%) than those reported previously.

It is therefore likely that by altering the Li:Al:P ratios we have modified the glassy network of Al–O and P–O units, creating more favorable pathways for Li-ion transport. No significant differences were observed in XPS spectra, and limited structural information could be obtained by XRD (Figure S3 in the SI). Local structure studies using pair-distribution-function analysis and solid-state NMR techniques may help in better understanding these materials but are out of the scope of the present study.

### 3.6 Electronic Conductivity

**Figure 5:**
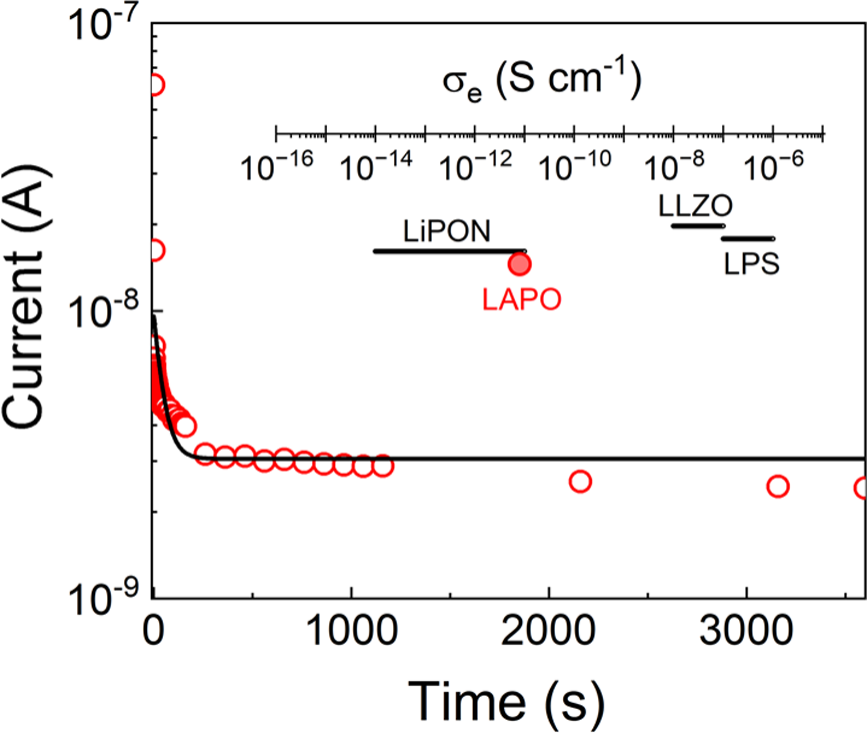
Current–voltage decay curve for a Li_2.8_AlP_1.25_O_*x*_ film annealed at 275 °C. Comparison of the σ_e_ of LAPO against SEs in the literature (inset).

The bulk electronic conductivity (σ_e_) of SEs has been suggested to be an important factor in the prevention of Li dendrites.^14^ To determine the σ_*e*_, a DC voltage was applied and the subsequent current decay monitored (Figure 5). From this steady-state current value, the σ_*e*_ of LAPO was calculated as ≈10^–11^ S cm^–1^, ≈4 orders of magnitude lower than the σ_*ion*_ and yielding a transference number of ≈1 assuming only the Li-ions are mobile. This value for LAPO compares well to that reported for LiPON (≈10^–11^–10^–14^ S cm^–1^)^14,53^ and is significantly lower than those for Li_7_La_3_Zr_2_O_12_ (LLZO) and Li_3_PS_4_ (LPS) SEs (Figure 5, inset).

### 3.7 Stability against Li Metal

**Figure 6:**
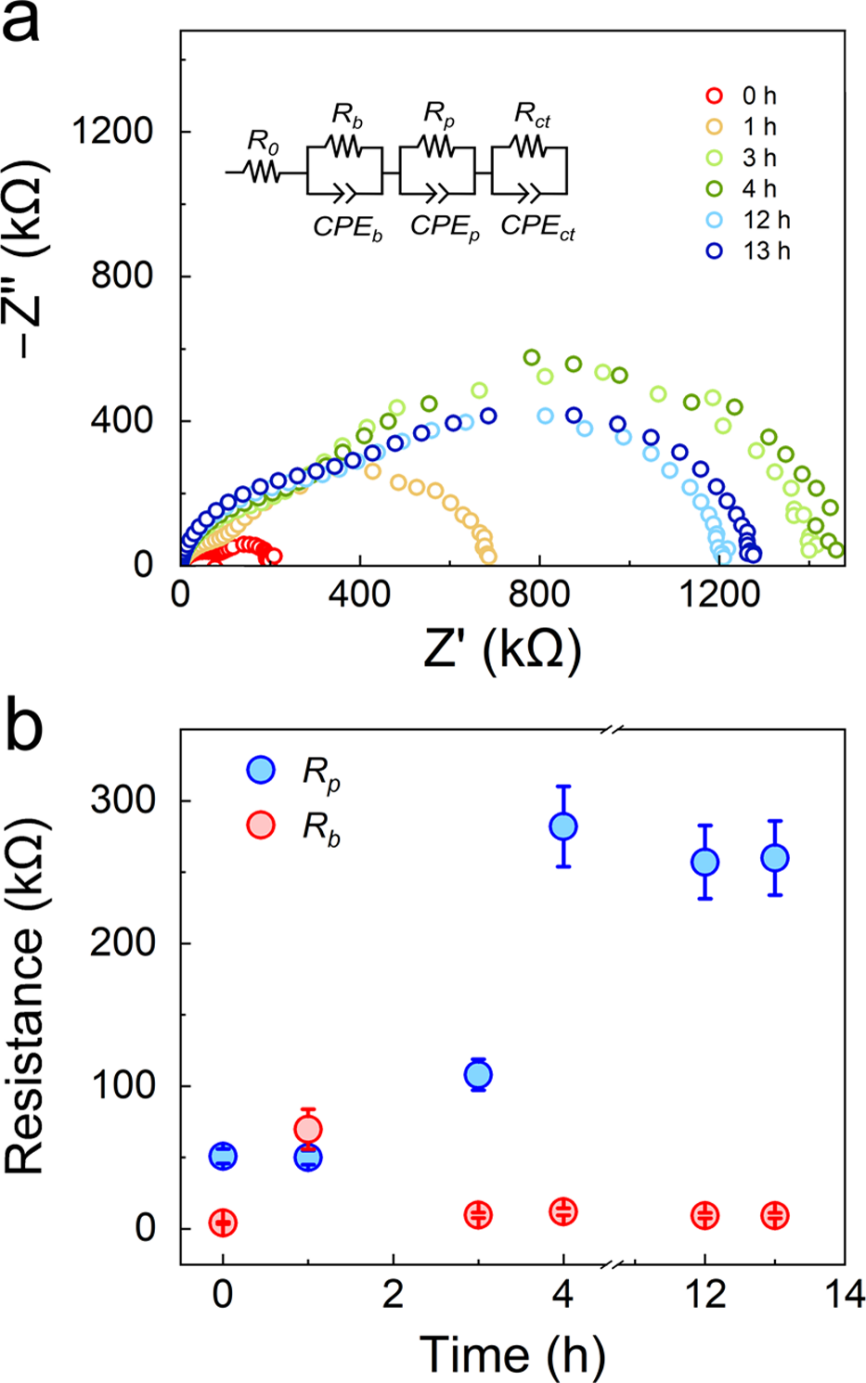
(a) EIS Nyquist spectra of Li_2.8_AlP_1.25_O_*x*_ against Li metal over 13 h in a Li|LAPO|Si configuration. (b) Comparison of *R*_b_ and *R*_p_ vs time. The resistance values were extracted from DRT analysis (Figure S7 in the SI).

The electrochemical stability of LAPO was tested against Li metal using time-dependent EIS (Figure 6a and Figure S6 in the SI). The EIS data were fit using 3 *R*|*CPE* units^54^ (ECM inset in Figure 6a). Here, *R*_1_ represented the bulk SE impedance (*R*_*b*_), *R*_2_ the passivation layer (*R*_*p*_) due to LAPO decomposition, and *R*_3_ the charge transfer at the Li interface (*R*_*ct*_). Distribution of relaxation times (DRT) analysis^44,55^ was used to deconvolute the different polarization processes. The characteristic frequencies (time constants) for *R*_*b*_, *R*_*p*_, and *R*_*ct*_ were ≈50 kHz (20 μs), ≈5 kHz (0.2 ms), and ≈100–1000 Hz (1–10 ms), respectively. To test the linearity, stability, and causality of the EIS data, the Kramers–Kroning relation was first applied.^40^ The residuals were fixed to be ±1% for the processes occurring at high-to-mid frequencies corresponding to the passivation and bulk SE resistances *R*_*b*_ and *R*_*p*_, respectively. However, at mid-to-low frequencies the DRT residuals were outside the set range, possibly due to the non-linear nature of the charge transfer reactions occurring at the Li interface.

Therefore, it was not possible to meaningfully quantify the *R*_*ct*_ values. The non-linearity of the *R*_*ct*_ and shift in time processes can be observed in the DRT plot (Figure S7 in the SI). Figure 6b shows that *R*_*b*_ was fairly invariant with time, with an anomaly at 1 h which we attribute to the decomposition reaction of LAPO with Li. A stable passivation layer or interphase was formed after ≈4 h with an ≈25× greater impedance than the bulk SE. Reactivity with Li is true of most SEs, with only LLZO and LiPON forming stable, low impedance interphases against Li to the best of our knowledge.^3,4^ That the native LAPO–Li interphase is stable is promising for future surface modification and composition engineering efforts to reduce or eliminate impedance due to the formed interphase.

### 3.8 In Situ XPS during Li Deposition

**Figure 7:**
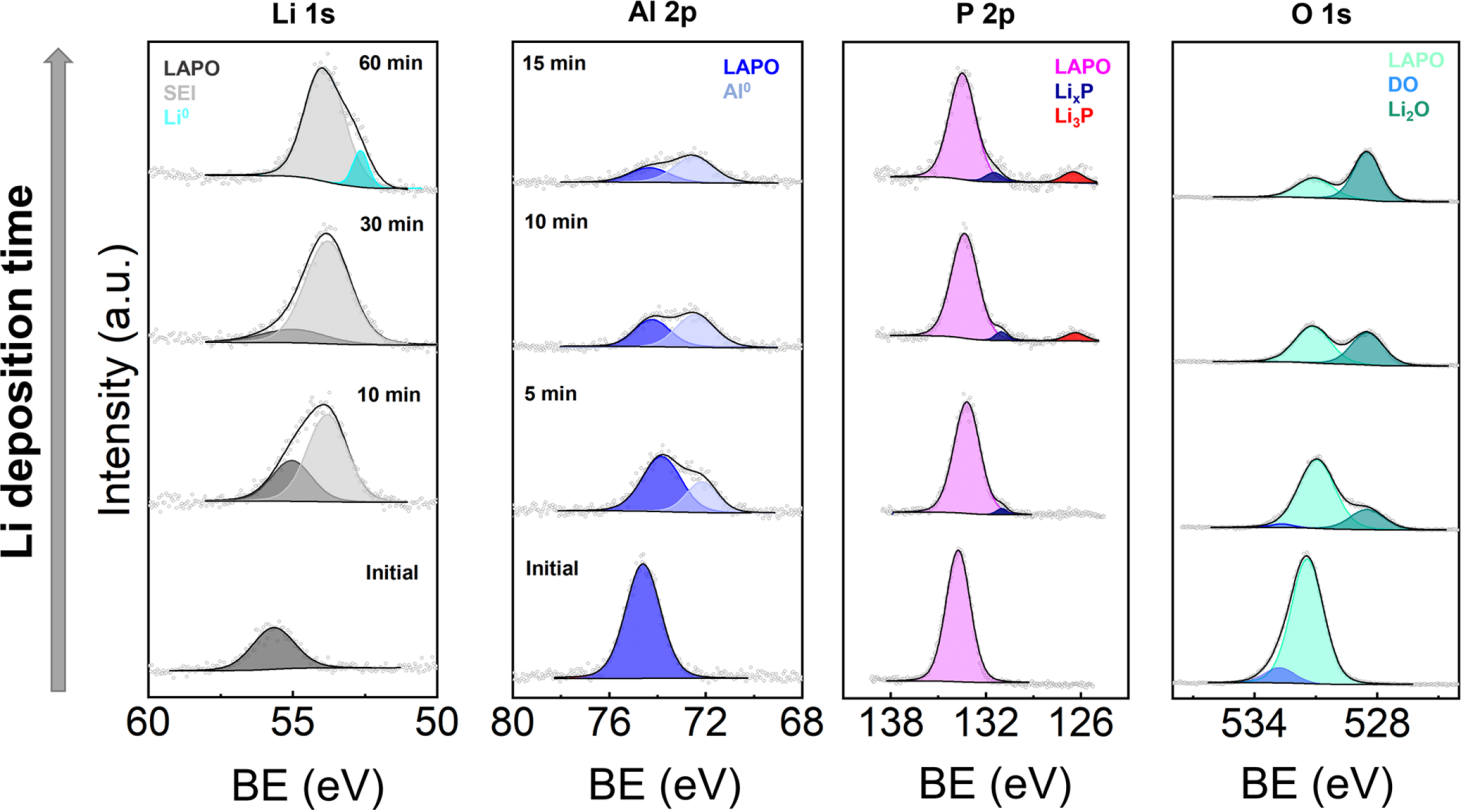
Evolution of core level XPS spectra during Li deposition on the Li_2.8_AlP_1.25_O_*x*_ surface. Note that the deposition times given for the Al 2p spectra also apply for P 2p and O 1s panels.

To elucidate the interphase composition, we performed in situ XPS, monitoring the core-level photoemission spectra Li 1s, Al 2p, P 2p, and O 1s during Li deposition (Figure 7). In all cases, the pristine components remained in addition to new features caused by reaction with Li, suggesting either that LAPO exists as part of the passivation layer or that this layer is thin enough to permit sampling of the underlying SE. Note that the initial XPS data were charge corrected to adventitious carbon (C 1s). However, upon Li deposition this feature is no longer reliable as a reference point; therefore, the Li_2_O peak in the O 1s spectrum was used instead.^37^ This disparity was responsible for the initial shift in binding energy (BE). Thereafter, small (<0.5 eV) shifts in BE were a result of the changing chemical environments for each element as decomposition continued.

In the case of Li 1s, a new feature appeared at lower BE (≈53.7 eV). This was likely due to the formation of Li-containing decomposition products, such as Li_2_O, Li_3_P, Li_*x*_P, and surface-absorbed contaminants such as Li_2_CO_3_ etc. After 60 min, an additional peak at lower BE emerged (≈52.7 eV) associated with Li^0^. A significant fraction of the Al^3+^ initially present was reduced to Al^0^ during Li deposition. Neither the Al 2p or Li 1s spectra displayed the energy shifts associated with Li–Al alloying,^56^ while P^5+^ was reduced to Li_3_P (126.5 eV) and partially reduced Li_*x*_P species (131.0 eV). A very similar evolution of the P 2p spectra was observed during in situ XPS of LiPON.^57^ Finally, a new feature at lower BE was detected in the O 1s spectra, which grew to dominate with time and could be assigned to Li_2_O. Although there will be some Li_2_O present due to the deposited Li reacting with surface contaminants and trace O_2_/H_2_O present inside the XPS chamber,^58^ it is likely that a majority of the Li_2_O formed as a result of direct reaction with LAPO considering the greater impedance of the interphase. Therefore, the passivation layer was found to be a mixture of Li_2_O, Li_3_P, Li_*x*_P,and Al^0^ species. A stable interphase should contain ionically conducting and electronically insulating decomposition products.^59^ Considering the chemical information from XPS and the resistive interphase revealed by EIS, we speculate that the electrically conductive components (Li_3_P, Li_*x*_P, Al^0^) were isolated in a matrix of Li_2_O, which is a known electronic insulator.^60^

## 4 Conclusions

In summary, non-crystalline Li-ion SE thin films were synthesized from aqueous solutions. Through systematic exploration of the Li–Al–P–O phase space, an optimal composition of Li_2.8_AlP_1.25_O_*x*_ was identified with an σ_*ion*_ > 10^–7^ S cm^–1^ at room temperature. Both increased Li and decreased P contents were required to maximize. Higher annealing temperatures led to decreased σ_*ion*_ between 230 and 400 °C in this system, despite remaining X-ray amorphous at all temperatures studied. Film surface roughness exhibited a complex dependence on annealing temperature, with the smoothest films being produced at 275 °C. Temperature-dependent σ_*ion*_ measurements yielded a low activation energy of 0.42(1) eV, indicating facile Li-ion transport in this SE. DC polarization experiments revealed a low σ_*e*_ (≈10^–11^ S cm^–1^), and a moderate Young’s modulus of ≈54 GPa was also determined. In contact with Li metal, LAPO formed a stable but resistive passivation layer, and in situ XPS showed this to consist of Li_2_O, Li_3_P, Li_*x*_P, and Al^0^ species. These findings should motivate future investigations into solution-processed non-crystalline SEs to further improve their bulk and interfacial properties for use in advanced energy storage devices.

## Supporting Information Available

Room temperature ionic conductivity of lithium aluminophosphate films, lab GIXRD, XPS region scans, additional activation energy analyses, EIS, and DRT (PDF)
