# Supplementary material for: Engineering Solution-Processed Non-Crystalline Solid Electrolytes for Li Metal Batteries
Source: Chem Mater. 2023 Jan 18;35(3):1168–76. doi: 10.1021/acs.chemmater.2c03071 (PMC9933431; doi:10.1021/acs.chemmater.2c03071)
Supplement: Supplementary file 1 — cm2c03071_si_001.pdf [file cm2c03071_si_001.pdf]

# Supporting Information for:

# Engineering Solution-Processed Non-Crystalline Solid Electrolytes for Li Metal Batteries

Pooja Vadhva,[a] Thomas E. Gill,[a] Joshua H. Cruddos,[a,b] Samia Said,[a] Marco Siniscalchi,[c] Sudarshan Narayanan,[b,c] Mauro Pasta,[b,c] Thomas S. Miller,[a,b] Alexander J. E. Rettie[a,b,*]

[a] Electrochemical Innovation Lab, Department of Chemical Engineering, University College London, WC1E 6DH (UK)

[b] The Faraday Institution Quad One, Harwell Science and Innovation Campus, Didcot OX11 0RA (UK)

[c] Department of Materials, University of Oxford, OX1 3PH (UK)

**Table S1.** Comparison of ionic conductivity conducted at $T$ (K) of Li-Al-P-O bulk glasses and solution processed thin films.[1–4]

| Composition | Processing type | $T$ (K) | $\sigma_{ion}$ (S cm$^{-1}$) | Reference |
|---|---|---|---|---|
| $Li_{2.8}AlP_{1.25}O_x$ | Solution | 295 | $1.8\times10^{-7}$ | This study |
| $Li_{2.5}AlP_{1.5}O_x$ | Solution | 295 | $2.6\times10^{-8}$ | Clayton et al., 2017 |
| $Li_{2.5}AlP_{1.5}O_{6.5}$ | Melt | 303 | $2.8\times10^{-8}$ | Reddy et al., 2012 |
| $Li_{2.33}AlP_5O_{15.67}$ | Melt | 403 | $2.1\times10^{-7}$ | Sharma et al., 2009 |
| $Li_{10}AlP_9O_{29}$ | Melt | 295 (extrapolated) | $1.3\times10^{-8}$ | Moreau et al., 2009 |

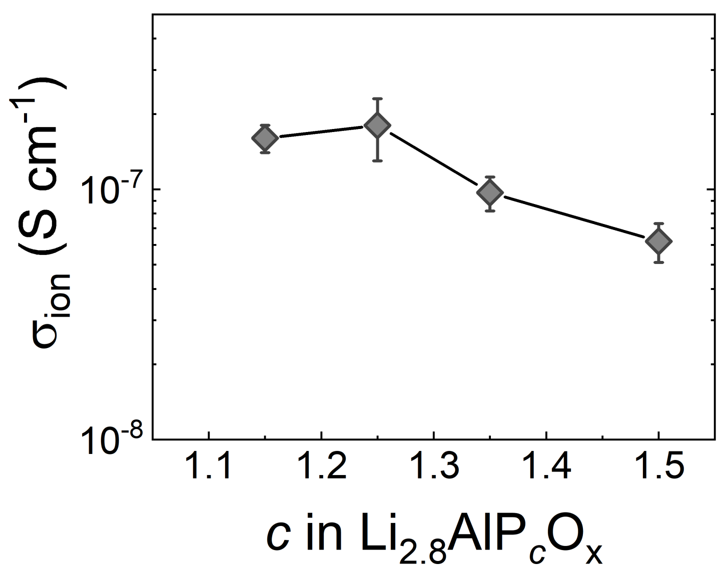


**Figure S1.** Ionic conductivity for $Li_{2.8}AlP_cO_x$ films determined from the fitted EIS data using the ECM displayed in Figure 1a in the main text.

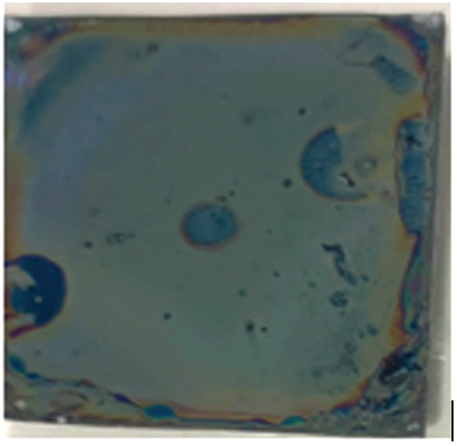

**Figure S2.** Photograph of a LAPO film annealed at 200 °C on a 2×2 cm$^2$ Si wafer.

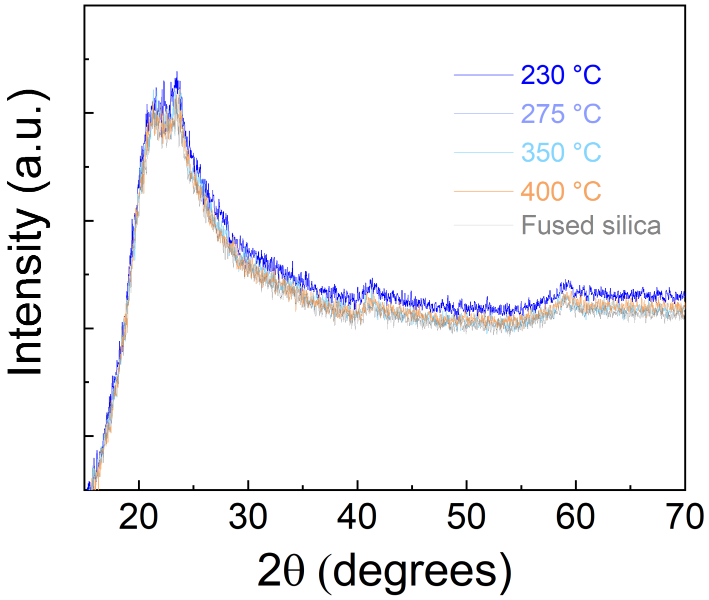


**Figure S3.** Theta-theta XRD on LAPO films annealed at 230, 275, 350 and 400 ºC. No detectable signal from the films is seen above that from the fused silica substrate.

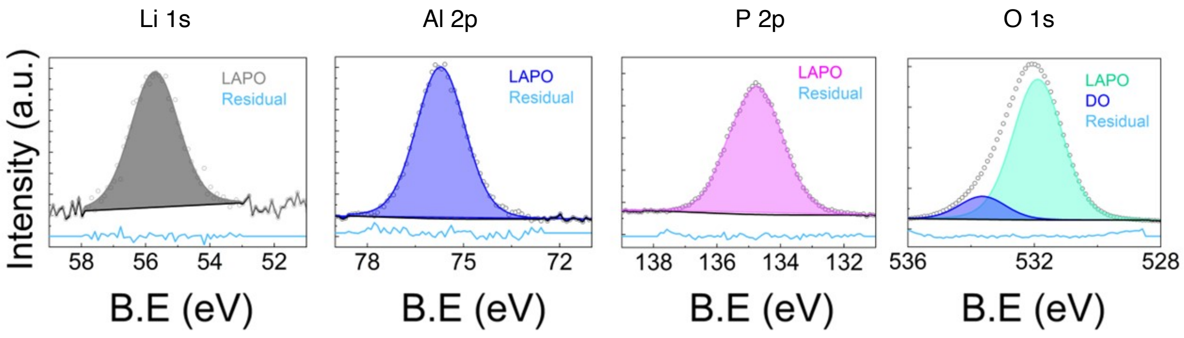


**Figure S4.** Representative XPS region spectra for a $Li_{2.8}AlP_{1.25}O_x$ film.

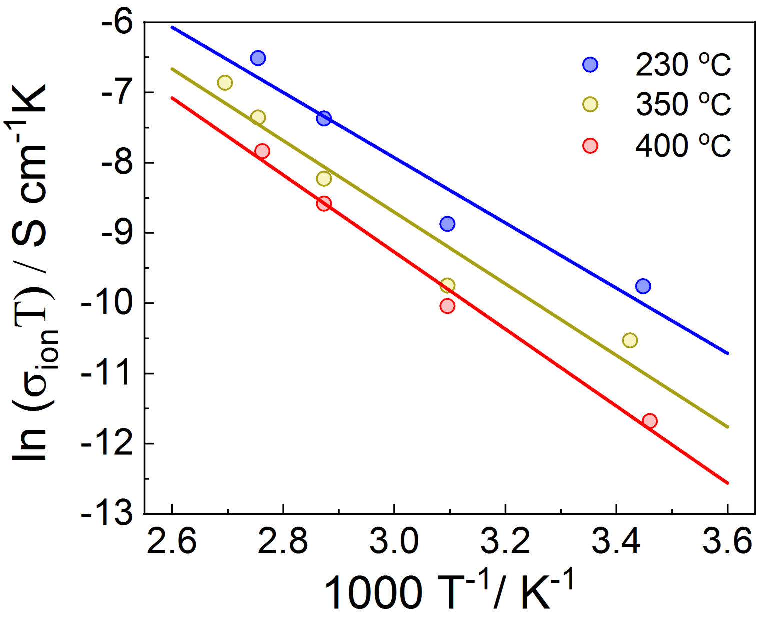


**Figure S5.** Temperature-dependent ionic conductivity measurements and activation energy analysis on $Li_{2.8}AlP_{1.25}O_x$ films annealed at 230, 350 and 400 °C.

**Table S2.** Calculated activation energies of $Li_{2.8}AlP_{1.25}O_x$ films annealed at different temperatures (Figure S5). The temperature-dependent ionic conductivity data for the film annealed at 275 °C is shown in the main text (Figure 4).

| $T_{anneal}$ (°C) | Activation Energy (eV) |
|---|---|
| | $E_a$ (eV) |
| 230 | 0.39(7) |
| 275 | 0.42(1) |
| 350 | 0.44(6) |
| 400 | 0.47(3) |

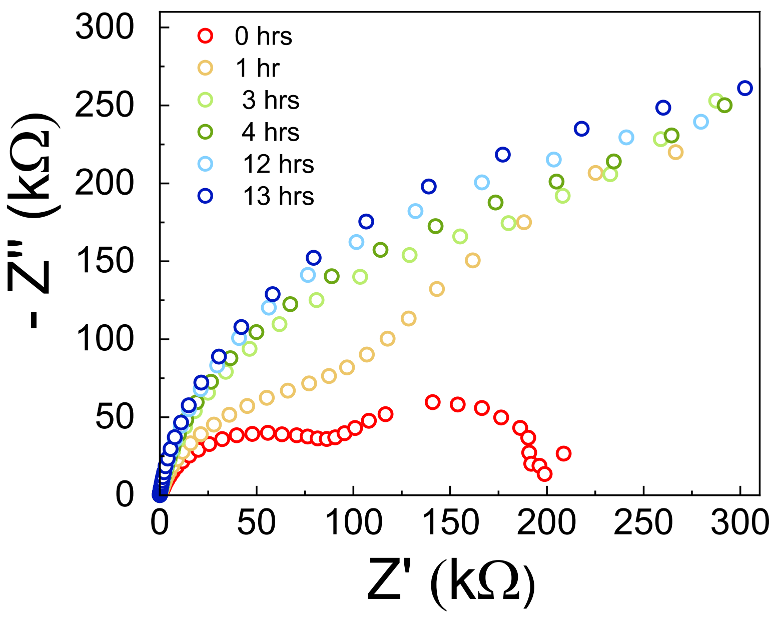


**Figure S6.** Zoomed in plot of EIS data showing features between 0 to 3 h.

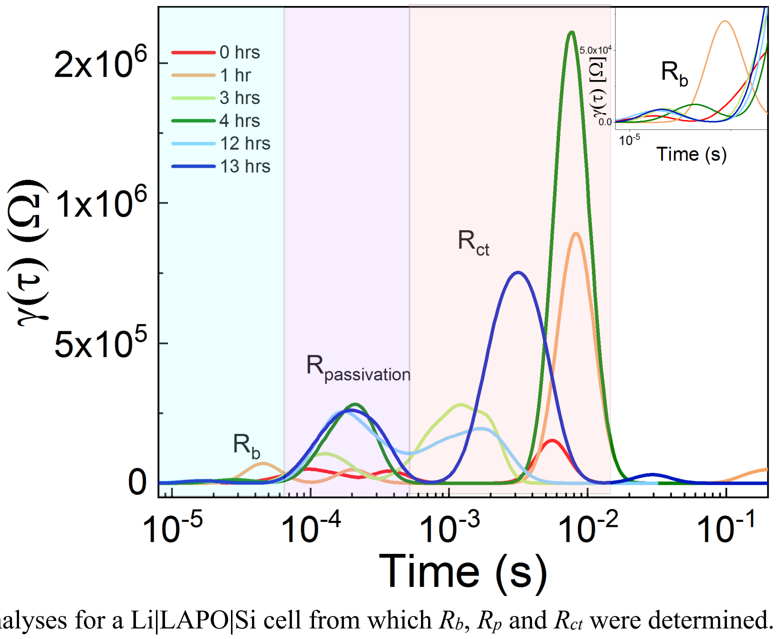


**Figure S7.** DRT analyses for a Li|LAPO|Si cell from which $R_b$, $R_p$ and $R_{ct}$ were determined. The inset shows the $R_b$ at different times.

## References


1 D. R. Clayton, D. Lepage, P. N. Plassmeyer, C. J. Page and M. C. Lonergan, *RSC Adv.*, 2017, **7**, 7046–7051.

2 C. V. K. Reddy, R. B. Rao, K. C. Mouli, D. V. R. Koti Reddy and M. V. R. Reddy, *J. Mater. Sci.*, 2012, **47**, 6254–6262.

3 M. V. N. V. D. Sharma, A. V. Sarma and R. Balaji Rao, *J. Mater. Sci.*, 2009, **44**, 5557–5562.

4 F. Moreau, A. Durán and F. Muñoz, *J. Eur. Ceram. Soc.*, 2009, **29**, 1895–1902.
